# Contrast‐enhanced CT radiomics for preoperative evaluation of microvascular invasion in hepatocellular carcinoma: A two‐center study

**DOI:** 10.1002/ctm2.111

**Published:** 2020-06-21

**Authors:** Xiuming Zhang, Shijian Ruan, Wenbo Xiao, Jiayuan Shao, Wuwei Tian, Weihai Liu, Zhao Zhang, Dalong Wan, Jiacheng Huang, Qiang Huang, Yunjun Yang, Hanjin Yang, Yong Ding, Wenjie Liang, Xueli Bai, Tingbo Liang

**Affiliations:** ^1^ Department of Pathology, The First Affiliated Hospital, College of Medicine Zhejiang University Hangzhou China; ^2^ College of Information Science and Electronic Engineering Zhejiang University Hangzhou China; ^3^ Department of Radiology, The First Affiliated Hospital, College of Medicine Zhejiang University Hangzhou China; ^4^ Department of Radiology The People's Hospital of Beilun District Ningbo China; ^5^ Department of Radiology The First Affiliated Hospital, Wenzhou Medical University Wenzhou China; ^6^ Department of Hepatobiliary and Pancreatic Surgery, The First Affiliated Hospital Zhejiang University School of Medicine Hangzhou China; ^7^ Zhejiang Provincial Key Laboratory of Pancreatic Disease, The First Affiliated Hospital Zhejiang University School of Medicine Hangzhou China; ^8^ Innovation Center for the Study of Pancreatic Diseases, The First Affiliated Hospital Zhejiang University School of Medicine Hangzhou China

**Keywords:** contrast‐enhanced CT, hepatocellular carcinoma, microvascular invasion, multivariable logistic regression, radiomics

## Abstract

**Background:**

The present study constructed and validated the use of contrast‐enhanced computed tomography (CT)‐based radiomics to preoperatively predict microvascular invasion (MVI) status (positive vs negative) and risk (low vs high) in patients with hepatocellular carcinoma (HCC).

**Methods:**

We enrolled 637 patients from two independent institutions. Patients from Institution I were randomly divided into a training cohort of 451 patients and a test cohort of 111 patients. Patients from Institution II served as an independent validation set. The LASSO algorithm was used for the selection of 798 radiomics features. Two classifiers for predicting MVI status and MVI risk were developed using multivariable logistic regression. We also performed a survival analysis to investigate the potentially prognostic value of the proposed MVI classifiers.

**Results:**

The developed radiomics signature predicted MVI status with an area under the receiver operating characteristic curve (AUC) of .780, .776, and .743 in the training, test, and independent validation cohorts, respectively. The final MVI status classifier that integrated two clinical factors (age and α‐fetoprotein level) achieved AUC of .806, .803, and .796 in the training, test, and independent validation cohorts, respectively. For MVI risk stratification, the AUCs of the radiomics signature were .746, .664, and .700 in the training, test, and independent validation cohorts, respectively, and the AUCs of the final MVI risk classifier‐integrated clinical stage were .783, .778, and .740, respectively. Survival analysis showed that our MVI status classifier significantly stratified patients for short overall survival or early tumor recurrence.

**Conclusions:**

Our CT radiomics‐based models were able to predict MVI status and MVI risk of HCC and might serve as a reliable preoperative evaluation tool.

## Graphical abstract

(1) Preoperative computed tomography images of hepatocellular carcinoma (HCC) were collected from two institutions for training and independent validation.
(2) The least absolute shrinkage and selection operator regression algorithm was used to construct radiomics signatures.
(3) Radiomics‐based prediction models predicted the microvascular invasion status (positive vs. negative) and risk (low vs. high) of HCC.

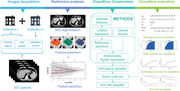

## 1 INTRODUCTION

Primary hepatocellular carcinoma (HCC) is a lethal disease that ranks fourth and third in China for morbidity and mortality, respectively.^1^ HCC not only threatens human health but also causes a heavy economic burden.^2^ Patients with HCC may undergo resection, liver transplantation, or radiofrequency ablation as a clinical cure based on the clinical stage. However, because of tumor recurrence, the 5‐year survival of HCC patients is still unsatisfactory.

A previous meta‐analysis results showed that microvascular invasion (MVI) of HCC is an indicator of great importance in long‐term survival and recurrence after surgical resection, serving as a hallmark of strong tumor invasion.^3^, ^4^ A series of studies have confirmed that the prognosis of patients with high‐risk MVI is worse than that of patients with low‐risk MVI after resection of HCC or liver transplant.^5^, ^6^ According to the 2017 edition guidelines for the diagnosis and treatment of liver tumors in China, pathological evaluations of positive MVI should be further classified as low or high risk based on the number and distribution of MVI.^7^ Therefore, a precise preoperative evaluation of MVI in HCC is needed to facilitate the establishment of individualized therapeutic schemes and surveillance strategies.

Previous studies have shown that some clinical and radiological characteristics are independent predictors for the MVI status of HCC.^4^, ^8^, ^9^, ^10^, ^11^, ^12^, ^13^, ^14^, ^15^ However, there is no widely accepted preoperative individualized predictor. Clinical factors, including age, tumor size/number, tumor differentiation, and serum levels of α‐fetoprotein (AFP) and antagonist‐II, are independent predictors of MVI and might be used for MVI prediction of HCC.^4^, ^8^, ^9^, ^10^, ^11^ Although these predictors exhibit various evaluation efficiencies, preoperative MVI evaluation of HCC is not available in clinical practice. Needle biopsy may be used for pathological diagnoses of hepatic tumors in specific situations, but effective MVI evaluation of HCC is unavailable due to the limited sample. Nonetheless, preoperative images have potential value for MVI prediction of HCC. Indeed, radiological characteristics of the capsule, irregular tumor margin, peritumoral enhancement, increased metabolism, and higher mean kurtosis value are useful for preoperative evaluations of MVI in HCC.^10^, ^12^, ^13^, ^14^, ^15^ Although these imaging characteristics are encouraging, they are not sufficient for individual evaluations of the preoperative MVI status of HCC.

A variety of artificial intelligence algorithms have recently been used for tumor evaluations. Radiomics is defined as the conversion of medical images into high‐throughput features to quantitatively evaluate tumor phenotypes.^16^ Numerous studies have shown that radiomics‐based models effectively predict the diagnosis, therapeutic efficacy, and prognosis of cancer patients for clinical decision‐making.^17^, ^18^, ^19^, ^20^, ^21^ Some progress in the MVI evaluation of HCC was recently made mainly using radiomics.^22^, ^23^, ^24^, ^25^, ^26^, ^27^ Most of these studies employed LASSO to select features, and logistic regression has achieved a combination of radiomics features and clinical factors. Regardless, these studies enrolled patients from a single institution, which may restrict the generalization of the radiomics model to other institution datasets. These studies also focused on predicting the MVI status of HCC, and further risk stratification for MVI‐positive HCC was lacking.

Therefore, more research data are required to obtain valuable models for multilevel MVI stratification in HCC to meet the individualized needs of clinical evaluation. The present study collected preoperative computed tomography (CT) images of HCC from two institutions as training and independent validation datasets for MVI prediction models. As far as we know, this is the first radiomics study to focus on the multilevel stratification of MVI in HCC patients.

## 2 METHODS

### 2.1 Study design

We enrolled patients from the First Affiliated Hospital, College of Medicine, Zhejiang University, Hangzhou (Institution I) and the First Affiliated Hospital of Wenzhou Medical University, Wenzhou (Institution II). The review boards of these two medical institutions approved the study protocol and waived the requirement of informed consent from patients. Patients from Institution I were randomly divided into a training cohort and a test cohort to construct and test the proposed classifiers. The dataset from Institution II served as the independent validation cohort.

**FIGURE 1:**
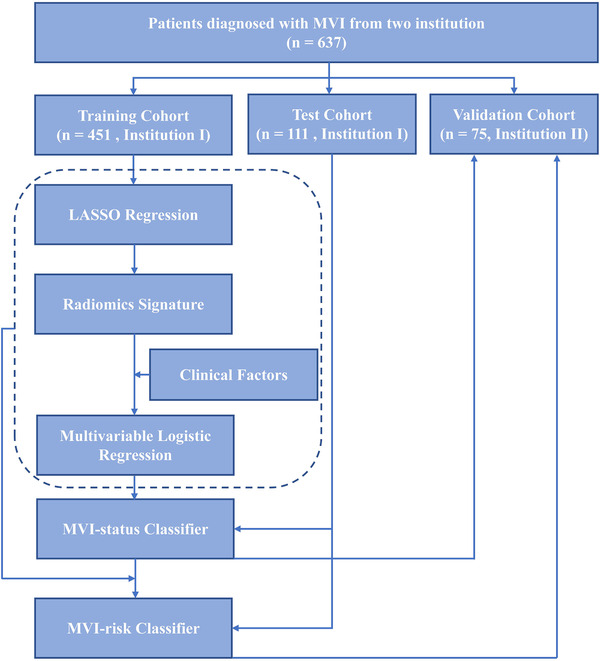
Workflow of this radiomics study

Two classifiers were constructed to predict MVI status and relevant risk. At the first level, a “signature” of MVI status was developed to predict the MVI status of patients with HCC. We scored MVI status using the classification results of radiomics signatures to reflect the possibility of assessing MVI in each patient. The final MVI status classifier was constructed by integrating the MVI status score and clinical factors. At the second level, we similarly developed a risk classifier for patients with MVI to predict MVI risk (high vs low). The constructed MVI status classifier and MVI risk classifier are represented as nomograms. A flowchart of our radiomics study is presented in Figure 1.

### 2.2 Inclusion criteria

The inclusion criteria were patients (a) with pathologically diagnosed HCC after surgical resection; (b) who preoperatively underwent contrast‐enhanced CT scan of the liver <1 month; and (c) with well‐preserved imaging data, clinical data, and pathological specimens for subsequent reevaluation.

### 2.3 Exclusion criteria

The exclusion criteria were patients (a) with recurrent HCC; (b) who underwent radiotherapy, chemotherapy, radiofrequency ablation, or other antitumor treatments before contrast‐enhanced CT scan; and (c) with poor imaging data that were unsuitable for region of interest (ROI) delineation.

### 2.4 Patient population

Patients in the two institutions who met the inclusion criteria were collected from March 2015 to March 2018. A total of 637 patients from the two institutions were recruited. The patient recruitment pathway is depicted in Figure S1. The overall patient imaging datasets were divided into three cohorts: training, test, and independent validation. The training cohort and test cohort were used for model training and test, respectively. The independent validation cohort was used for validating the proposed MVI classifiers. Clinical factors at baseline were collected for each patient, such as sex, age, tumor location (left lobe, right lobe, and caudate lobe), tumor number (single and multiple), maximum diameter, serum AFP, and clinical stage. A clinician with more than 5 years of clinical experience collected the data. The clinical stage of HCC (Ia/b, IIa/b, and IIIa/b) was evaluated according to the diagnosis and treatment guidelines for liver cancer.^7^ Our patients’ surgical procedures were developed in accordance with the guidelines for the diagnosis and treatment of liver cancer. We also attached importance to the multidisciplinary diagnosis and treatment model to avoid the limitations of a single discipline.

### 2.5 Pathological MVI evaluation

Two pathologists evaluated the MVI status of all HCC cases by observing hematoxylin eosin (HE)‐stained slices under the microscope. Positive MVI was defined as cancer cell nests in the vascular cavity under the microscope, primarily in portal vein branches. According to the guidelines for the diagnosis and treatment of liver cancer,^7^ the number and distribution of MVI were used for MVI risk classification. The three MVI risk levels included MVI negative, low‐risk MVI positive (no more than five MVI within 1 cm of tumor‐adjacent tissues), and high‐risk MVI positive (more than five MVI or MVI in nontumor‐adjacent tissues). If the MVI results were inconclusive, special staining was used to further identify the vessel walls, including CD34 (vascular endothelium), SMA (vascular smooth muscle layer), elastic fibers (miniature vessel wall elasticity fibrous layer), and D2‐40 (lymphatic endothelium).

### 2.6 CT imaging

Preoperative contrast‐enhanced CT of the liver was performed for each patient with HCC in this study at the two institutions. Institution I performed contrast‐enhanced CT using two CT scanners: a 16‐slice scanner (Aquilion; Toshiba Medical Systems, Tokyo, Japan) and a 256‐slice scanner (Brilliance iCT; Philips Healthcare, Cleveland, OH, USA). Institution II performed contrast‐enhanced CT using three CT scanners: a 320‐slice scanner (Aquilion ONE; Toshiba Medical Systems, Otawara, Japan), a 16‐slice scanner (BrightSpeed; GE Healthcare, Milwaukee, WI, USA), and a 2×64‐slice dual‐source scanner (Discovery CT750 HD; GE Healthcare, Milwaukee, WI, USA).

The CT parameters were tube voltage of 100 or 120 kVp, tube current of 200‐700 mAs, pixel spacing of 0.539‐0.881 mm, and slice thickness of 0.625‐5.000 mm. The nonionic contrast agents used were Iohexol (Yangtze River Pharmaceutical Group, Taizhou, China) and Iodixanol and Iohexol (GE Healthcare Ireland, Carrigtohill, Ireland). We used a high‐pressure syringe (3.0 mL/s) to inject nonionic contrast agent (1.5 mL/kg). CT scans were performed at 25‐35 s (arterial phase), 55‐75 s (portal‐vein phase), and 120‐180 s (delay phase) postinjection.

### 2.7 ROI segmentation

The delay phase data of contrast‐enhanced CT were used for our radiomics study. Because of the vital role of tumor‐adjacent tissues in the pathological evaluation of MVI,^28^ the tumor lesion and adjacent nontumor tissue were used as the ROIs in this study. A two‐step procedure was performed for ROI segmentation. First, the tumor lesion was only segmented manually on the cross‐sectional layer of the maximum tumor area using ITK SNAP (www.itksnap.org), with the consent of two radiologists. Second, tumor‐adjacent tissue was delineated automatically with a 1‐cm peritumoral border extension using “Dilation Operation” in MATLAB 2018b (MathWorks, Natick, MA, USA). Tumor‐adjacent tissue was further “retuned” to exclude surrounding organs, bones, and air via segmentation of the liver region. The liver area was segmented automatically using the “Fuzzy Clustering” algorithm.^29^ A senior radiologist checked all of the tumor segmentation results. Two cases of ROI segmentation are presented in Figure S2.

### 2.8 Radiomics feature extraction

Diversity in voxel sizes leads to variability in feature values.^30^ To select reproducible image features, a resampling strategy of voxel size is needed for medical images reconstructed at different voxel sizes.^31^ Therefore, we used spline interpolation to resample all images to an identical pixel size of 1 × 1 mm. The voxel intensities within the ROI were discretized to a limited intensity range of 64 bins.^32^

We extracted 798 radiomics features from the ROI for each case. The feature pool comprised seven intensity‐based features, the maximum diameter, 158 raw texture features, and 632 wavelet‐based texture features.^33^ Raw‐texture features were extracted from the gray‐level co‐occurrence matrix (GLCM), gray‐level run‐length matrix (GLRLM), gray‐level size zone matrix (GLSZM), and neighborhood gray‐tone difference matrix (NGTDM). Four types of GLCMs based on orientations (0°, 45°, 90°, and 135°) were considered at this point. Eighty‐eight GLCM features, 22 from each type of GLCM, were selected in our study. Regarding GLRLM features, 52 were extracted from the tumor region, with every 13 features from one type of GLRLM (run length = 1, 2, 3, and 4). The numbers of GLSZM‐ and NGTDM‐based features were 13 and 5, respectively. The tumor region was transformed to obtain 632 wavelet‐based texture features. All parameters of radiomics are described in Table S1.

### 2.9 Radiomics feature selection and construction of the MVI status signature

Each radiomics feature was normalized using the *Z*‐scores method to eliminate differences introduced by value scales between features. The features in the test and independent validation sets were normalized based on the mean value and standard deviation derived from the training cohort.

To construct a radiomics signature without overfitting, we used the least absolute shrinkage and selection operator (LASSO) regression algorithm to pick out the optimal contributing feature sets.^34^ LASSO regression regularized the feature set as follows:

$$\begin{array}{ccc} \hat{y} & = & \hat{\beta_{0}}\;+\hat{\beta_{1}}\times x_{1}+\hat{\beta_{2}}\times x_{2}+\cdots +\hat{\beta_{n}}\times x_{n},\\ \hat{\beta } & = & \;argmin\left\{{\left|y-X\beta \right|}^{2}+\lambda {\left|\beta \right|}_{1}\right\}, \end{array}$$

where *y* is the actual MVI status for each patient (0 for MVI negative; 1 for MVI positive), *x* is the individual feature, *β* is the LASSO coefficient, and *λ* is the penalty term. As parameter λ increases, *β* for each feature decreases.

Features with corresponding coefficients that were not zero in the LASSO regression results were retained. During the 100 iterations of LASSO, the AUC was calculated as the criterion with 10‐fold cross‐validation to select the optimal λ value. An MVI status signature was generated by summing the selected features weighted with respective coefficients, and an MVI status score was calculated to reflect the probability of MVI for each case. The predictive capability of the derived signatures was evaluated by the receiver operating characteristic (ROC) curves and the areas under ROC curves (AUCs).

### 2.10 Construction and evaluation of the MVI status classifier

A multivariable logistic regression (MLR) method was employed to implement the MVI status classifier by integrating the MVI status score and clinical factors. Clinical factors were age, sex, tumor location, maximum diameter, tumor number, AFP level, and clinical stage. Taking the minimum Akaike information criterion (AIC) index as the stop criterion, the backward search method was applied to determine the optimal combination. A model constructed with only clinical factors was used for comparison with the radiomics‐based MVI status classifier. A combination nomogram was created based on the proposed MVI status classifier.

The predictive accuracy of the MVI status classifier was evaluated using ROC curves and calibration curves. The latter were used to evaluate the difference between the predicted probability of MVI and the de facto MVI probability. The proximity degree between the diagonal and the calibration curve reflected the predictive accuracy of the models.

### 2.11 Construction and evaluation of the MVI risk classifier

To further stratify the risk of MVI, we developed a second classifier to predict it (high vs low) for patients with MVI. The methods for developing the MVI risk classifier were identical to the construction methods for the MVI status classifier. First, an MVI risk signature was developed using only screened radiomics features based on the LASSO feature‐selection method, and then an MVI risk score was computed to reflect a high MVI risk level for each patient. Second, the MVI risk classifier was constructed by combining the MVI risk signature and clinical factors using the MLR method.

### 2.12 Survival analyses

The present study employed the Kaplan‐Meier method for survival analysis to examine the prognostic value of the proposed two MVI classifiers. The follow‐up data recorded included total overall survival (OS) time and recurrence. Patients from Institution I and Institution II were assigned to the “MVI positive group” and “MVI negative group” or “high‐risk MVI group” and “low‐risk MVI group” based on the proposed MVI classifiers, which applied the threshold calculated in the training set according to the Youden Index. Log‐rank statistics were applied for group analysis of the survival curves.

### 2.13 Statistical analyses

The Mann‐Whitney *U*‐test or chi‐squared test was applied to univariate analyses of the clinical characteristics between groups with different MVI levels in the training set, test set, and independent validation set. The level of hypothesis testing was *P* < .05.

R v3.5.1 (www.R-project.org, 2016) was used for statistical analyses. The “glmnet” package in R software was employed for LASSO logistic regression. Calibration curves were drawn with the “rms” package, and nomograms for the developed classifiers were constructed with the “regplot” package.

## 3 RESULTS

### 3.1 Patient characteristics

In Institution I, 562 patients met the inclusion criteria, and these data were used to train and test models. For Institution I, 38.7% of the patients (n = 218) had MVI, and all were divided randomly into a training cohort (n = 451; 380 males and 71 females; MVI positive, 175; MVI negative, 276; age, 57.49 ± 10.82 years) and test cohort (n = 111; 102 males and nine females; MVI positive, 43; MVI negative, 68; age, 56.23 ± 11.16 years). In total, 75 of the patients in Institution II met the inclusion criteria and served as the independent validation cohort (63 males and 12 females; MVI positive, 37; MVI negative, 38; age, 60.71 ± 9.31 years). The detailed clinical characteristics of the patients with HCC are shown in Table 1. The distributions of MVI status, age, sex, tumor location, tumor maximum diameter, tumor number, and AFP level were not significantly different.

**TABLE 1:** Clinical characteristics of patients with HCC in the training cohort, test cohort, and validation cohort

Characteristics	Training cohort N = 451	Test cohort N = 111	*P*‐value	Validation cohort N = 75	*P*‐value
MVI status (Positive:Negative)	175:276	43: 68	.990	37: 38	.085
Age (year)	57.49 ± 10.82	56.23 ± 11.16	.138	60.71 ± 9.31	.033
Sex (Male:Female)	380:71	102:9	.056	63:12	.381
Tumor location (L:R)	133:318	30:81	.814	18:57	.468
Maximum diameter (cm)	5.04 ± 3.24	4.87 ± 3.09	.623	5.83 ± 4.35	.389
Tumor number (Single:Multiple)	414:37	104:7	.639	65:10	.221
Serum AFP level (Normal:Abnormal)	187:264	55:56	.152	36:39	.350
Clinical stage (T1a:Others)	309:142	83:28	.242	36:39	<.001

Abbreviations: AFP, alpha‐fetoprotein; L, the left lobe of liver; MVI, microvascular invasion; R, the right lobe of liver.

The clinical characteristics of the patients with different MVI statuses are provided in Table S2. The maximum diameter of tumors was significantly different between the MVI‐positive and MVI‐negative patients in the training, test, and independent validation datasets. In addition, the proportion of patients with an abnormal AFP level in the MVI‐positive group was significantly higher in the training and test cohorts. Clinical stage was significantly different for the training cohort and independent validation cohort (*P* < .05) in MVI status. The clinical characteristics of the low‐risk MVI and high‐risk MVI patients are shown in Table S3. A significant difference between the low‐risk MVI and high‐risk MVI patients in two cohorts was only found for the maximum tumor diameter (*P* < .05).

### 3.2 Radiomics feature selection and construction of the MVI status signature

**FIGURE 2:**
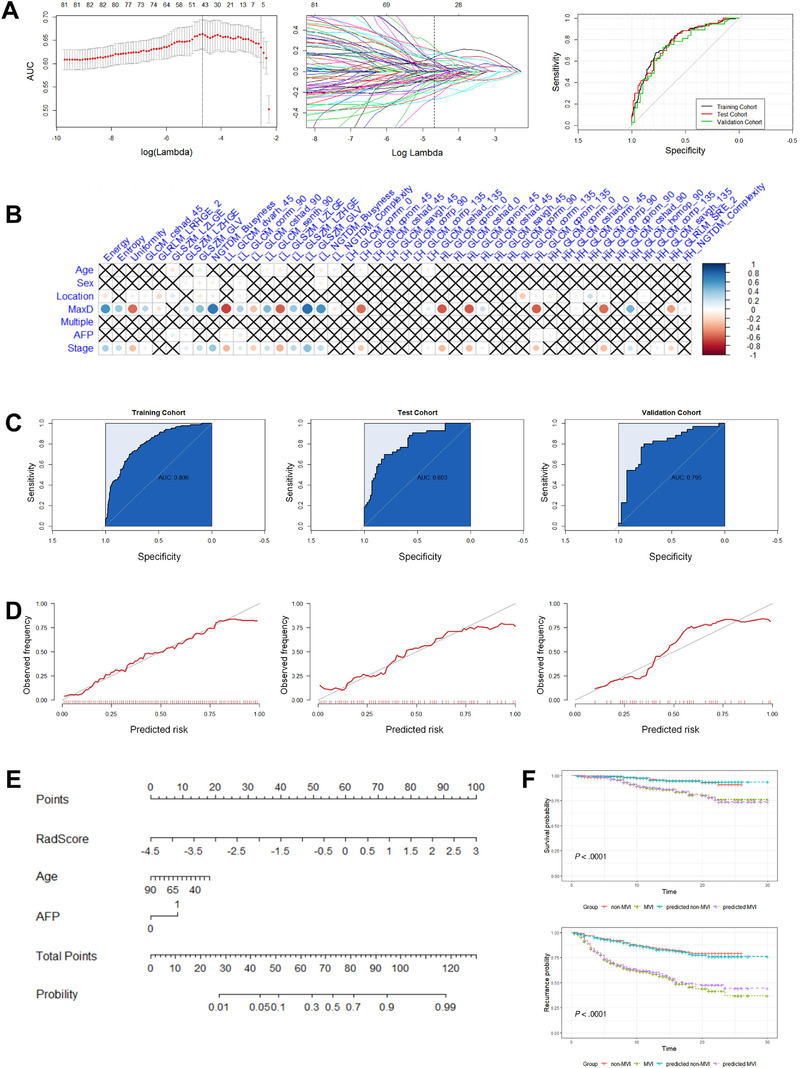
Results of constructing the MVI status classifier. A, Radiomics feature selection and receiver operating characteristic curves for the MVI status signature in the training, test, and independent validation cohorts. (Left) Cross‐validation AUC plot at different λ values. The first dotted line shows the location of the highest AUC and best λ value. (Middle) Coefficients of each feature in the LASSO feature selection process at different λ values. The dotted line shows the feature coefficient at the best λ value. (Right) ROC curves for the MVI status signature in the training, test, and validation cohorts. B, Correlation plot of clinical factors and selected radiomics features for constructing the MVI status signature. C, ROC curves for the MVI status classifier in the training, test, and validation cohorts. D, Calibration curves for the MVI status classifier in the training, test, and validation cohorts. E, Nomogram for the MVI status classifier incorporating the α‐fetoprotein (AFP) level, age (Age), and radiomics signature (RadScore). F, Survival analyses using the known MVI status and predicted MVI status. (Top) Survival analyses for patients with known MVI status (MVI positive vs MVI negative, *P* < .001) and predicted MVI status (predicted MVI positive vs predicted MVI negative, *P* < .001). (Bottom) Recurrence analyses for patients with known MVI status (MVI positive vs MVI negative, *P* < .001) and predicted MVI status (predicted MVI positive vs predicted MVI negative, *P* < .001)

We extracted 798 radiomics features from each ROI image; 30 were excluded because of near‐zero variance. The intercorrelation matrix was constructed, and 685 radiomics features were found to be highly correlated (correlation coefficient > .75), leaving 83 radiomics features for LASSO‐based feature selection. The model showed the highest mean AUC in 10‐fold cross‐validation when *λ* was set as .008416903. Forty‐four radiomics features with coefficients that were not zero were selected. An MVI status signature was constructed according to the selected radiomics features and corresponding coefficients in the LASSO process. The detailed formula of the MVI status score is shown in Formula S1. According to the radiomics signature, the AUC was .780 (95% confidence interval [CI], .736‐.823) in the training set (Figure 2A). Consistent prediction performances were also observed in the test (AUC: .776; 95% CI, .688‐.864) and independent validation (AUC: .743; 95% CI, .630‐.856) cohorts.

### 3.3 Construction and evaluation of the MVI status classifier

The multivariable model combining the MVI status signature, age, and AFP level, which was the ideal MVI status classifier, showed the smallest AIC (Table S4). The MVI status classifier resulted in an AUC of .806 (95% CI, .769‐.849) in the training cohort, .803 (95% CI, .725‐.890) in the test cohort, and .796 (95% CI, .693‐.905) in the validation cohort (Figure 2C). For the model constructed with only clinical factors, the AUC was .827 (95% CI, .727‐.927) in the training cohort, .771 (95% CI, .679‐.862) in the test cohort, and .739 (95% CI, .692‐.786) in the validation cohort. Analyses of the calibration curves revealed good calibration between the classifier‐predicted MVI probability and the actual probability of MVI (Figure 2D). The radiomics model is illustrated as a nomogram in Figure 2E.

### 3.4 Construction and evaluation of the MVI risk classifier

**FIGURE 3:**
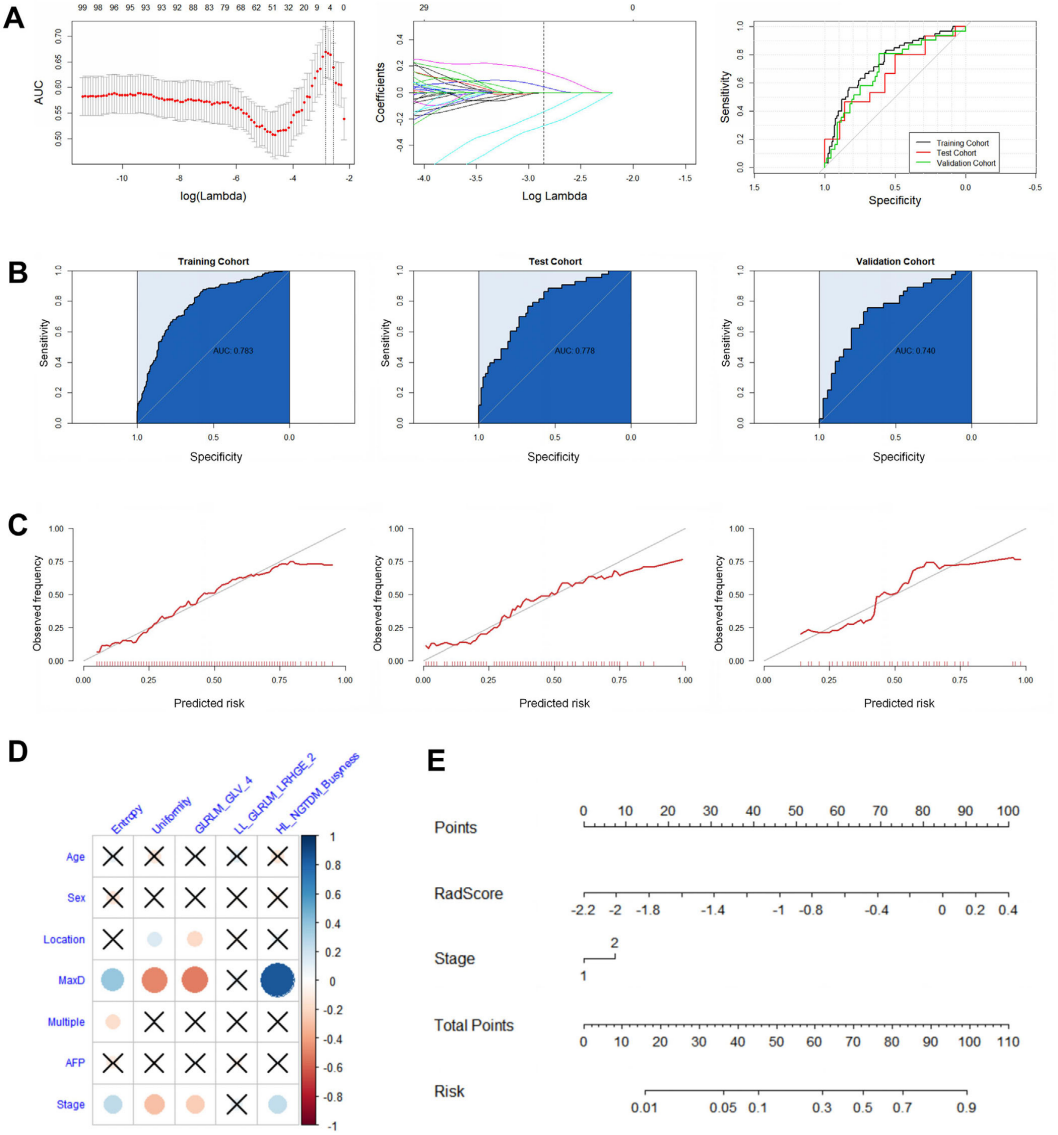
Results of constructing the MVI risk classifier. A, Radiomics feature selection and receiver operating characteristic curves for the MVI risk signature in the training, test, and independent validation cohorts. (Left) Cross‐validation AUC plot at different λ values. The first dotted line shows the location of the highest AUC and best λ value. (Middle) Coefficients of each feature in the LASSO feature selection process at different λ values. The dotted line shows the feature coefficient at the best λ value. (right) ROC curves for the MVI risk signature in the training, test, and validation cohorts. B, ROC curves for the MVI risk classifier in the training, test, and validation cohorts. C, Calibration curves for the MVI risk classifier in the training, test, and validation cohorts. D, Correlation plot of clinical factors and selected radiomics features for constructing the MVI risk signature. E, Nomogram for the MVI risk classifier incorporating clinical stage (Stage) and radiomics signature (RadScore)

LASSO selected five radiomics features to construct the MVI risk signature at *λ* = .05765982. The detailed formula of the MVI risk score is shown in Formula S2. In the training cohort, the signature revealed an AUC of .746 (95% CI, .670‐.823). The AUC based on the signature in the test cohort was .664 (95% CI, .487‐.842), and that in the validation cohort was .700 (95% CI, .586‐.813) (Figure 3A). The combination of the MVI risk signature and clinical stage showed the smallest AIC, and it was used as the MVI risk classifier for predicting MVI risk (Table S5). The model showed an AUC of .783 (95% CI, .740‐.826) in the training cohort, .778 (95% CI, .691‐.866) in the test cohort, and .740 (95% CI, .627‐.854) in the validation cohort (Figure 3B). According to the model that used only clinical factors to predict MVI risk, the AUC was .742 (95% CI, .665‐.818) in the training cohort, .719 (95% CI, .553‐.885) in the test cohort, and .529 (95% CI, .335‐.724) in the validation cohort. The calibration curves also indicated good calibration between classifier‐predicted high‐risk MVI probability and the actual high‐risk MVI probability (Figure 3C). The nomogram for MVI risk stratification is presented in Figure 3E. The performance comparison of different models using the independent validation cohort is provided in Table S6.

### 3.5 Survival analyses

Follow‐up information on OS time, recurrence status, and recurrence time was available for 565 patients in Institutions I and II. Forty‐five of the patients died during the follow‐up. The average duration of follow‐up was 12.5 months. A total of 151 patients experienced tumor recurrence during the follow‐up. Moreover, OS and recurrence were significantly different between the MVI‐positive group and MVI‐negative group (*P *< .001) based on our MVI status classifier, which supports the prognostic stratification value of the model (Figure 2F). There was no significant difference in survival or recurrence between the predicted high‐risk MVI group and low‐risk MVI group (Figure S3).

## 4 DISCUSSION

We constructed two classifiers in the present study: one classifier was used to preoperatively evaluate the MVI status of HCC patients and the other to further stratify MVI risk. The MVI status classifier that combined radiomics signatures with age and AFP level showed AUCs of .806 for the training cohort, .803 for the test cohort, and .796 for the validation cohort. The combination of the MVI risk signature and clinical stage as the ideal MVI risk classifier had an AUC of .783 for the training set, 0.778 for the test set, and .740 for the validation set. Validation of the constructed MVI classifiers from independent institutions confirmed the generalizability of the developed multilevel MVI classifiers. The radiomics signature of the MVI status classifier is also a potential imaging biomarker. Finally, we review the findings of recent radiomics‐based studies for MVI stratification in Table S7.^23^, ^25^, ^26^, ^27^, ^35^, ^36^, ^37^, ^38^, ^39^

Our study results confirmed that wavelet transformation considerably contributes to radiomics‐based models for MVI evaluations of HCC. A previous study found that a radiomics model based on CT showed good predictive accuracy for MVI status of hepatitis B virus‐related HCC patients.^25^ The constructed radiomics signature for predicting MVI status included eight radiomics features selected from texture and shape features. However, our study included these original radiomics features and wavelet‐based features in the radiomics signatures, and the latter showed higher weight coefficients in our final models. Our results are consistent with a recent study that used a gadoxetic acid‐enhanced MRI‐based radiomics method to predict MVI status in HCC.^35^ The Long Run High Gray‐level Emphasis (LRHGE) feature derived from the GLRLM of the tumor region was valuable in our CT‐based study and in an MRI‐based radiomics study.^35^ Based on the results, GLRLM_LRHGE may be an important cross‐modality radiomics feature for predicting MVI status.

Plasma AFP levels may play a significant role in the fused model for MVI status prediction of HCC. In fact, McHugh and colleagues showed that the MVI status of HCC was significantly associated with AFP levels.^9^ According to another study, the plasma AFP level is an independent predictor that can be used to construct a preoperative MVI prediction model of HCC.^11^ Consistent with these results, we demonstrated plasma AFP levels to be significantly different between the two MVI status groups of HCC. In addition, the AFP level was recently used to construct an MVI prediction model based on radiomics.^23^, ^25^, ^26^, ^27^ Nevertheless, the threshold of AFP level varies in different radiomics nomograms for MVI evaluation, which may lead to the inapplicability of these models in real clinical situations. Our study incorporated qualitative evaluation results of serum AFP levels into our predictive model, facilitating clinical use.

No consensus has been reached on with regard to whether tumor size is an independent predictive factor in MVI evaluation models for HCC. Previous studies have reported that tumor size between MVI‐positive group and MVI‐negative group of HCC is significantly different, and that tumor size may be used to construct MVI prediction models.^8^, ^9^, ^10^, ^11^ Two radiomics studies that assessed MVI of HCC also found tumor size to be an independent clinical factor for the construction of radiomics nomograms.^23^, ^27^ Although the different MVI status groups in our study had distinct tumor sizes, the inclusion of tumor size did not improve the predictive effectiveness of the radiomics model. We performed correlation analyses between clinical factors and selected radiomics features, and most of the selected radiomics features of the radiomics signature correlated significantly with tumor size (Figures 2B and 3D); thus, the role of tumor size was replaced in the prediction model. Accordingly, tumor size was not included in our final MVI prediction models of HCC.

Our study did not incorporate the clinical stage of HCC in the MVI status classifier, but it did play a role in MVI risk prediction. In most radiomics studies for MVI prediction of HCC, the clinical stage of HCC has not been investigated,^23^, ^24^, ^25^, ^27^ even though Xu and colleagues demonstrated the importance of clinical stage in their MVI status model of HCC.^26^ However, in our study, clinical stage failed to improve the predictive efficacy of the MVI status model of HCC, partially because clinical staging correlates strongly with the specific radiomics features (Figure 2B). Notably, the clinical stage of HCC improved the predictive efficacy of the MVI risk classifier for HCC, but four of the five selected radiomics features correlated highly with the clinical stage (Figure 3D). From these investigative results, we infer that clinical stage played a unique role in the MVI risk model. Therefore, the clinical stage of HCC was included as an independent factor in our radiomics model for MVI risk stratification.

There are a few limitations in our radiomics study. First, the morphologic features of HCC were not evaluated because we investigated the efficacy of the MVI prediction model based on objective quantitative radiomics features. We will further explore the evaluation efficacy of a fused model that includes radiomics signatures and morphological features. Second, the use of two‐dimensional (2D) ROIs is another possible shortcoming of our radiomics research because information on three‐dimensional (3D)‐segmented tumors is more abundant. We will attempt to compare the prediction performance of MVI evaluation models based on 2D and 3D imaging data in future studies. Third, the overall follow‐up time was relatively short because we included HCC cases that met the pathological criteria in recent years. We will continue to follow up with the enrolled patients.

## 5 CONCLUSIONS

Our radiomics models based on a CT radiomics signature and clinical factors realized multiple levels of MVI stratification and useful for preoperative evaluation of HCC. Our MVI evaluation models are potential prognostic imaging markers for postoperative patients with HCC.

## CONFLICT OF INTEREST

The authors declare no conflict of interest.

## Supporting information

Supporting Information
