# Supplementary material for: Contrast‐enhanced CT radiomics for preoperative evaluation of microvascular invasion in hepatocellular carcinoma: A two‐center study
Source: Clin Transl Med. 2020 Jun 21;10(2):e111. doi: 10.1002/ctm2.111 (PMC7403665; doi:10.1002/ctm2.111)
Supplement: Supplementary file 1 — Supporting Information [file CTM2-10-e111-s001.docx]

## Supplementary

**Table S1:** Details of the extracted radiomics features.

| **Feature Category** | | | **Feature List** |
| --- | --- | --- | --- |
| Global Features | | Shape | Maximum Diameter |
|  |  | Intensity-based Features | (1) Uniformity; (2) Variance; (3) Energy;(4) Skewness; (5) Entropy; (6) Kurtosis; (7) Mean; |
| Raw  Texture Features | Gray-level co-occurrence matrix (GLCM) features  (0^o^,45^o^,90^o^,135^o^) | | (1) Energy [energ]; (2) Correlation 2 [corrp]; (3) Sum of Square Variance [sosvh]; (4) Homogeneity 2 [homop]; (5) Autocorrelation [autoc]; (6) Maximum Probability [maxpr]; (7) Entropy [entro]; (8) Homogeneity 1 [homom]; (9) Sum Variance [svarh]; (10) Cluster Prominence [cprom]; (11) Dissimilarity [dissi]; (12) Correlation 1 [corrm]; (13) Information Measure of Correlation 1 [inf1h];(14) Difference Entropy [denth]; (15) Sum Entropy [senth];(16) Contrast [contr]; (17) Difference Variance [dvarh]; (18) Sum Average [savgh]; (19) Inverse Difference Normalized [indnc]; (20) Inverse Difference Moment Normalized [idmnc]; (21) Cluster Shade [cshad];(22) Information Measure of Correlation 2 [inf2h]; |
|  | e.g. GLCM_cshad_45 | | The Cluster Shade derived from the 45^o^ GLCM of the tumor region. |
|  | Gray-level run-length matrix (GLRLM) features  (1,2,3,4) | | (1) Gray-level Non-uniformity [GLN];(2) Low Gray-level Run Emphasis [LGRE]; (3) Run Percentage [RP]; (4) Run-length Non-uniformity [RLN]; (5) High Gray-level Run Emphasis [HGRE];(6) Long Run Low Gray-level Emphasis [LRLGE]; (7) Gray-level Variance [GLV];(8) Long Run Emphasis [LRE]; (9) Run-length Variance [RLV]; (10) Short Run Low Gray-level Emphasis [SRLGE];(11) Long Run High Gray-level Emphasis [LRHGE]; (12) Short Run Emphasis [SRE]; (13) Short Run High Gray-level Emphasis [SRHGE]; |
|  | e.g. GLRLM_LRHGE_2 | | The Long Run Low Gray-level Emphasis derived from the length-2 GLRLM of the tumor region. |
|  | Gray-level size zone matrix (GLZSM) features | | (1) Gray-level Non-uniformity [GLN]; (2) High Gray-level Zone Emphasis [HGZE];(3) Small Zone Emphasis [SZE]; (4) Small Zone Low Gray-level Emphasis [SZLGE]; (5) Large Zone Emphasis [LZE]; (6) Zone Percentage [ZP]; (7) Zone-size Non-uniformity [ZSN];(8) Small Zone High Gray-level Emphasis [SZHGE]; (9) Large Zone High Gray-level Emphasis [LZHGE]; (10) Low Gray-level Zone Emphasis [LGZE];(11) Zone-size Variance [ZSV]; (12) Gray-level Variance [GLV]; (13) Large Zone Low Gray-level Emphasis [LZLGE]; |
|  | e.g. GLSZM_GLV | | The Gray-level Variance derived from the GLSZM of the tumor region. |
|  | Neighborhood gray-tone difference matrix (NGTDM) features | | (1) Strength; (2) Busyness; (3) Complexity; (4) Contrast; (5) Coarseness; |
|  | NGTDM_Busyness | | The Busyness derived from the NGTDM of the tumor region. |
| Wavelet-based texture features | | | 1. HL-; (2) HH-; (3) LL-; (4) LH-; |
| e.g. LL_GLCM_dvarh_45 | | | The Difference Variance derived from the 45^o^ GLCM of the tumor region transformed by low-pass filter on the x-axis and low-pass filter on the y-axis. |

| Characteristics | Training Cohort | |  | Test Cohort | |  | Validation Cohort | |  |
| --- | --- | --- | --- | --- | --- | --- | --- | --- | --- |
| MVI Status | MVI-negative | MVI-positive | *p* | MVI-negative | MVI-positive | *p* | MVI-negative | MVI-positive | *p* |
|  | 276 | 175 |  | 68 | 43 |  | 38 | 37 |  |
| Age (year)  median (range) | 60  (28-86) | 54  (25-80) | <0.001 | 58  (28-85) | 54  (32-75) | 0.066 | 64  (42-78) | 58  (45-88) | 0.069 |
| Sex  Male: Female | 229: 47 | 151: 24 | 0.418 | 63:5 | 39:4 | 0.992 | 32: 6 | 31: 6 | 1.000 |
| Tumor Location  L: R | 85:191 | 48:127 | 0.510 | 18:50 | 12:31 | 1.000 | 9:29 | 9:28 | 1.000 |
| Maximum Diameter (cm)  median (range) | 3.5  (0.5-18.0) | 5.5  (1.0-18.0) | <0.001 | 3.5  (1.2-9) | 6.0  (1.5-15.0) | <0.001 | 2.9  (0.5-11.6) | 7.0  (1.2-24.0) | <0.001 |
| Tumor Number  Single: Multiple | 255:21 | 159:16 | 0.687 | 66:2 | 38:5 | 0.152 | 35:3 | 30:7 | 0.287 |
| Serum AFP Level  Normal: Abnormal | 139:137 | 48:127 | <0.001 | 40:28 | 15: 28 | 0.023 | 22:16 | 14:23 | 0.094 |
| Clinical Stage  T1a: Others | 207: 69 | 102: 73 | <0.001 | 54: 14 | 29: 14 | 0.234 | 25: 13 | 11: 26 | 0.004 |

**Table S2:** Clinical characteristics for MVI-positive and MVI-negative patients with HCC in the training, test and validation cohorts.

**Note:** MVI, microvascular invasion; AFP, serum alpha-fetoprotein; L, the left lobe of liver; R, the right lobe of liver. Continuous variables, including age and maximum diameter, were analyzed using the Mann-Whitney test. The chi-squared test was used for other variables.

| Characteristics | Training | | | Testing | | |
| --- | --- | --- | --- | --- | --- | --- |
| MVI Risk | **Low-risk** | **High-risk** | *p* | **Low-risk** | **High-risk** | *p* |
|  | 115 | 60 |  | 28 | 15 |  |
| Age (year)  median (range) | 54  (25-80) | 55  (30-73) | 0.794 | 55  (32-68) | 51  (31-75) | 0.789 |
| Sex  Male: Female | 101:14 | 50:10 | 0.556 | 25:3 | 14:1 | 1.000 |
| Tumor Location  L: R | 31:84 | 17:43 | 0.988 | 9:19 | 3:12 | 0.625 |
| Maximum Diameter (cm)  median (range) | 5.0  (1.0-18.0) | 7.0  (2.0-16.0) | 0.004 | 4.0  (1.5-13.0) | 10.0  (4.0-15.0) | <0.001 |
| Tumor Number  Single: Multiple | 104:11 | 55:5 | 1.000 | 25:3 | 13:2 | 1.000 |
| Serum AFP Level  Normal: Abnormal | 34:81 | 14:46 | 0.485 | 12:16 | 3:12 | 0.245 |
| Clinical Stage  T1a: Others | 75:40 | 27:33 | 0.016 | 21:7 | 8:7 | 0.269 |

**Table S3:** Clinical characteristics for low-risk MVI and high-risk MVI patients with HCC in the training and test cohorts.

**Note:** MVI, microvascular invasion; AFP, serum alpha-fetoprotein; L, the left lobe of liver; R, the right lobe of liver. Continuous variables, including age and maximum diameter, were analyzed using the Mann-Whitney test. The chi-squared test was used for other variables.

**Table S4:** Selection process for the MVI-status prediction model.

| **Feature set** | **AIC** |
| --- | --- |
| RadScore + Age + Sex + Location + MaxD + Number + AFP + Stage | 510.61 |
| RadScore + Age + Sex + Location + MaxD + AFP + Stage | 508.61 |
| RadScore + Age + Sex + Location + MaxD + AFP | 507.05 |
| RadScore + Age + Sex + MaxD + AFP | 505.84 |
| RadScore + Age + MaxD + AFP | 504.80 |
| RadScore + Age + AFP | 504.01 |

**Note:** AIC, Akaike information criterion; RadScore, MVI status-score; MaxD, maximum diameter; Number, tumor number; AFP, serum AFP level; Stage, clinical stage.

**Table S5:** Selection process for the MVI-risk prediction model.

| **Feature set** | **AIC** |
| --- | --- |
| RadScore + Age + Sex + Location + MaxD + Number + AFP + Stage | 225.61 |
| RadScore + Sex + Location + MaxD + Number + AFP + Stage | 223.61 |
| RadScore + Sex + Location + Number + AFP + Stage | 221.61 |
| RadScore + Sex + Number + AFP + Stage | 219.65 |
| RadScore + Sex + Number + Stage | 218.05 |
| RadScore + Sex + Stage | 217.17 |
| RadScore + Stage | 216.27 |

**Note:** AIC, Akaike information criterion; RadScore, MVI status-score; MaxD, maximum diameter; Number, tumor number; AFP, serum AFP level; Stage, clinical stage.

**Table S6:** Performance comparison of different models in the independent validation set.

| **Index** | **MVI status** | | |  | **MVI risk** | | |
| --- | --- | --- | --- | --- | --- | --- | --- |
|  | **Clinical model** | **Radiomics signature** | **Nomogram** |  | **Clinical model** | **Radiomics signature** | **Nomogram** |
| Sensitivity | 0.461 | 0.625 | 0.708 |  | 0.358 | 0.643 | 0.650 |
| Specificity | 0.849 | 0.697 | 0.754 |  | 0.702 | 0.663 | 0.695 |
| AUC  (95% CI) | 0.739  (0.692-0.786) | 0.743  (0.630-0.856). | 0.796  (0.693-0.905) |  | 0.529  (0.335-0.724) | 0.700  (0.586-0.813) | 0.740  (0.627-0.854) |

**Note:** MVI, microvascular invasion; AUC, area under the curve; CI, confidence interval.

**Table S7:** Advancements and details in the classification of modeling methods, imaging modality, feature type, MVI stratification, independent validation set (with or without) and AUC in each study through radiomics.

| **Author** | **Modeling Algorithm** | **Imaging** | **Feature Signature** | **MVI**  **Stratification** | **Independent Validation Set** | **AUC** |
| --- | --- | --- | --- | --- | --- | --- |
| Zheng J [23] | logistic regression | CT | 16 ACM / 21 LBP features | 2 | Without | 0.770-0.940 |
| Peng J [25] | LASSO regression | CT | 8 radiomics features ( histogram, texture, shape) | 2 | Without | 0.774-0.915 |
| Xu X [26] | adjusted odds-ratio (OR) regression and logistic regression | CT | 8 features (5 radiographic features and 3 radiomics score) | 2 | Without | 0.851-0.919 |
| Ma XH [27] | SVM, LASSO regression and logistic regression | CT | 8 texture features (4 PVP texture features and 4 clinical features ) | 2 | Without | 0.672-0.930 |
| Ni M [35] | LASSO，GBDT | CT | / | 2 | Without | 0.880 |
| Hu HT [36] | LASSO regression and logistic regression | Ultrasound | 6 Radiomics features (texture, Laplacian of Gaussian features) | 2 | Without | 0.647-0.815 |
| Yao Z [37] | iterative SR method and SVM | Ultrasound | / | 2 | Without | 0.930-0.990 |
| Feng ST [38] | LASSO regression and logistic regression | MRI | 10 texture features | 2 | Without | 0.710-0.950 |
| Zhu YJ [39] | logistic regression | MRI | 4 AP features / 5 PP features  ( histogram and texture features) | 2 | Without | 0.706/0.794 |
| Our study | LASSO regression and logistic regression | CT | 44 Radiomics feature (MVI status) /  5 Radiomics feature (MVI risk) | 3 | With | 0.693-0.905 |

**Note:** CT, computed tomography; MRI, magnetic resonance imaging; AUC, area under the curve; ACM, angle cooccurrence matrices; LBP, local binary patterns; LASSO, least absolute shrinkage and selection operator; SVM, support vector machine; MVI, microvascular invasion; SR, superresolution; GBDT, gradient boosting decision tree; AP, arterial phase; PP/PVP, portal venous phase.

**Figure S1**

**
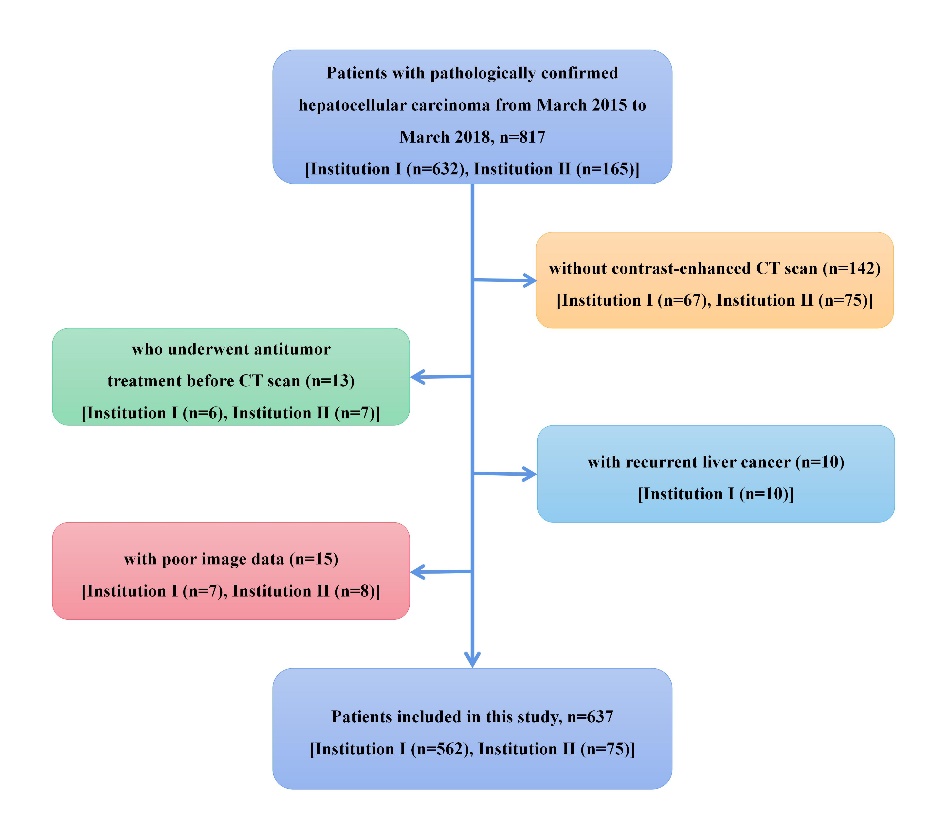
**

Figure S1. Patient recruitment pathway.

**Figure S2**

**
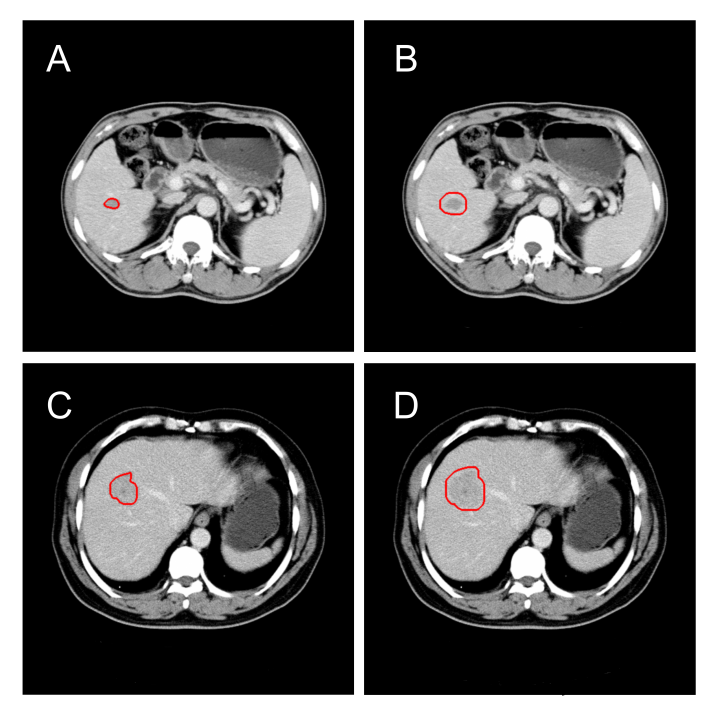
**

Figure S2. Two cases of ROI segmentation. (A) and (C) are the segmentations for tumor lesions. (B) and (D) are the segmentations for tumor lesions accompanied by tumor-adjacent tissue.

**.**

**Figure S3**


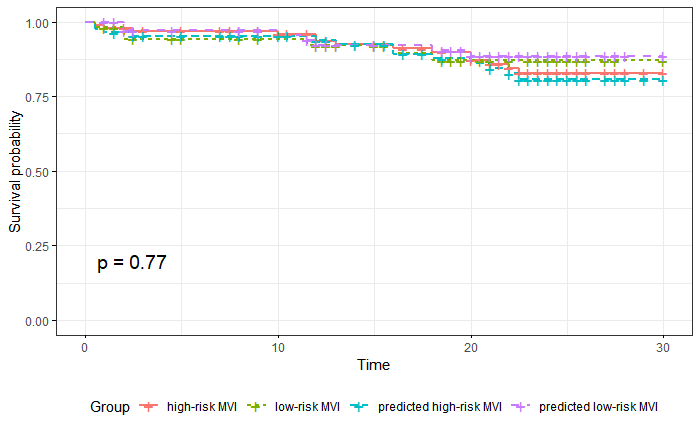

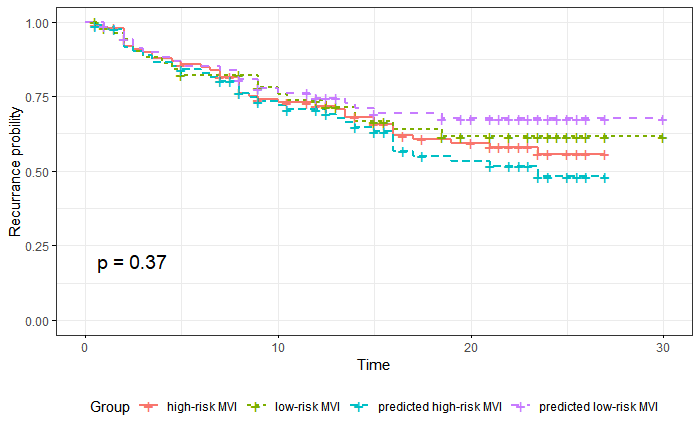

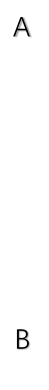


Figure S3. (A) Survival analysis for patients with known MVI risk (high-risk MVI *vs.* low-risk MVI) and predicted MVI risk (predicted high-risk MVI vs. predicted low-risk MVI). (B) Recurrence analysis for patients with known MVI risk (high-risk MVI *vs.* low-risk MVI) and predicted MVI risk (predicted high-risk MVI *vs.* predicted low-risk MVI).

**Formula S1**

$$MVI Status˗score= -0.553220835+0.069198318\times Intensity\_Energy+0.213914932\times Intensity\_Entropy - 0.086099208\times Intensity\_Uniformity + 0.115077693\times GLCM\_cshad\_45 -0.077807247\times GLRLM\_LRHGE\_2 + 0.127678228\times GLSZM\_LZLGE + 0.058463981 \times GLSZM\_GLV +0.004814754\times NGTDM\_Busyness -0.193204223 \times LL\_GLCM\_dvarh\_45+0.124092834\times LL\_GLCM\_corrm\_90+0.058038467\times LL\_GLCM\_cshad\_90+0.263205468\times LL\_GLCM\_senth\_90+0.037646816\times LL\_GLSZM\_LZLGE-0.007296215\times LL\_GLSZM\_LZHGE+0.054531294\times LL\_GLSZM\_GLV+0.166743529\times LL\_NGTDM\_Busyness-0.276029015\times LL\_NGTDM\_Complexity-0.074454272\times LH\_GLCM\_corrm\_0-0.04758729\times LH\_GLCM\_cprom\_45-0.003178869\times LH\_GLCM\_cshad\_45-0.22038699\times LH\_GLCM\_savgh\_45+0.039823713\times LH\_GLCM\_corrp\_90-0.271022728\times LH\_GLCM\_corrp\_135-0.082058353\times LH\_GLCM\_cshad\_135+0.123317778\times HL\_GLCM\_cprom\_0+0.119561178\times HL\_GLCM\_cshad\_0-0.069560662\times HL\_GLCM\_cprom\_45+0.021895002\times HL\_GLCM\_cshad\_45-0.102331706\times HL\_GLCM\_savgh\_45-0.064222801\times HL\_GLCM\_corrm\_90-0.166699076\times HL\_GLCM\_corrm\_135+0.10988692\times HL\_GLCM\_cprom\_135+0.201249878\times HH\_GLCM\_corrm\_0-0.037840439\times HH\_GLCM\_cshad\_0+0.115959428\times HH\_GLCM\_corrm\_45-0.14969525\times HH\_GLCM\_corrp\_90-0.074642408\times HH\_GLCM\_cprom\_90-0.081112888\times HH\_GLCM\_cshad\_90-0.113052037\times HH\_GLCM\_homop\_90-0.153319804\times HH\_GLCM\_corrp\_135-0.040744606\times HH\_GLCM\_savgh\_135-0.048516296\times HH\_GLRLM\_SRE\_2-0.035356862\times HH\_NGTDM\_Complexity$$

**Formula S2**

$$MVI Risk˗score= -0.6701899784+0.0449167176\times Intensity\_Entropy-0.2522478123\times Intensity\_Uniformity -0.0006592444\times GLCM\_GLV\_4 -0.1452383167\times LL\_GLRLM\_LRHGE\_2 +0.1554547206\times HL\_NGTDM\_Busyness$$
